# Lactylation-Associated Immune Metabolic Reprogramming Identifies *S100A2* and *S100A14* as Candidate Diagnostic Biomarkers in Primary Open-Angle Glaucoma: An Integrated Bulk and Single-Cell Transcriptomic Analysis

**DOI:** 10.3390/genes17040403

**Published:** 2026-03-31

**Authors:** Yu Xu, Xin Fu, Yajun Gong, Fangyuan Zeng, Min Tang, Sixian Hu, Guangyi Huang, Tianxian Tu, Xiaolai Zhou

**Affiliations:** Zhongshan Ophthalmic Center, Sun Yat-sen University, Guangzhou 510623, China; xuyu29@mail2.sysu.edu.cn (Y.X.); fx001225@163.com (X.F.); gyj08236@163.com (Y.G.); zfy0824@yeah.net (F.Z.); cc610375273@163.com (M.T.); hsx062584@163.com (S.H.); hgy0004@126.com (G.H.); ttx0128@126.com (T.T.)

**Keywords:** primary open-angle glaucoma, lactylation, transcriptomics, machine learning, immune microenvironment, single-cell RNA sequencing

## Abstract

**Background**: Primary open-angle glaucoma (POAG) is a leading cause of irreversible blindness worldwide, characterized by progressive optic nerve degeneration and marked molecular heterogeneity. Increasing evidence indicates that metabolic dysregulation and immune remodeling contribute to POAG pathogenesis; however, the underlying regulatory networks and reliable diagnostic biomarkers remain incompletely defined. **Methods**: Bulk transcriptomic and single-cell RNA sequencing (scRNA-seq) datasets of trabecular meshwork tissues were retrieved from Gene Expression Omnibus (GEO). Differential expression analysis and weighted gene co-expression network analysis (WGCNA) were performed to identify disease-associated modules. A machine learning framework was applied to construct classification models. Estimated immune-cell fractions were assessed using CIBERSORT, followed by pathway and transcription factor analyses. Single-cell analysis was conducted to examine the cell type-specific expression patterns. **Results**: A total of 195 differentially expressed genes were identified between POAG and control samples. WGCNA revealed a lactylation-related module strongly correlated with disease status. Machine learning identified *S100A2* and *S100A14* as candidate diagnostic biomarkers with consistent classification performance across datasets. Immune infiltration analysis suggested alterations in the immune microenvironment in POAG. Single-cell data showed that the model genes exhibited sparse but non-uniform expression across cell populations. **Conclusions**: This integrative analysis prioritizes *S100A2* and *S100A14* as candidate diagnostic biomarkers for POAG and indicates potential associations with immune-metabolic regulatory mechanisms.

## 1. Introduction

Primary open-angle glaucoma (POAG) is a chronic, progressive optic neuropathy and represents the leading cause of irreversible blindness worldwide [1]. It is characterized by the gradual loss of retinal ganglion cells and optic nerve damage, often accompanied by elevated intraocular pressure (IOP), although disease progression may continue despite effective IOP control [2]. Epidemiological studies estimate that the global number of individuals affected by glaucoma will exceed 110 million by 2040, highlighting its growing public health burden [3].

Accumulating evidence indicates that POAG is a multifactorial disease involving oxidative stress, extracellular matrix remodeling of the trabecular meshwork, immune dysregulation, and metabolic abnormalities rather than a purely mechanical disorder driven by IOP elevation alone [4,5]. In particular, immune-mediated neuroinflammation has emerged as a key contributor to optic nerve degeneration in glaucoma, with both innate and adaptive immune responses implicated in the progression of the disease [6,7].

Recent advances in high-throughput transcriptomic profiling have enabled systematic characterization of molecular alterations in glaucomatous tissues [8]. Bulk transcriptomic studies have revealed extensive gene expression remodeling in the trabecular meshwork and optic nerve head, providing insights into disease-associated pathways [9]. However, bulk analyses are inherently limited by cellular heterogeneity and cannot fully resolve cell type-specific regulatory programs. In this context, single-cell RNA sequencing (scRNA-seq) has emerged as a powerful approach to overcome these limitations by revealing previously unrecognized cellular diversity and distinct transcriptional states, thereby providing a higher-resolution framework for elucidating the molecular mechanisms underlying glaucoma [10,11].

In parallel, increasingly appreciated is the importance of metabolic regulation in disease-associated transcriptional control. At the epigenetic level, histone lysine lactylation, a recently identified post-translational modification derived from cellular metabolism, provides a mechanistic link between metabolic state and gene regulation [12]. Although lactylation has been implicated in immune regulation and disease pathogenesis in multiple systems, its potential involvement in glaucoma-related transcriptional networks remains largely unexplored [13].

Machine learning approaches have increasingly been applied to ophthalmic research and have demonstrated strong potential for disease classification and biomarker discovery in glaucoma [14]. By integrating transcriptomic features with advanced computational modeling, machine learning provides a powerful framework for identifying candidate diagnostic markers from high-dimensional biological data.

In the present study, we applied an integrative analytical framework combining bulk transcriptomics, weighted gene co-expression network analysis, immune deconvolution, machine learning-based classification modeling, and single-cell contextual analysis to systematically investigate lactylation-associated molecular signatures and their diagnostic relevance in POAG. This multi-layered approach aimed to identify potential diagnostic biomarkers and to provide a more comprehensive view of the immune and metabolic dysregulation underlying POAG pathogenesis.

Therefore, the present study integrates lactylation-associated genes with diagnostic modeling and cellular resolution validation to investigate molecular heterogeneity in POAG. Given the limited understanding of lactylation-associated regulatory mechanisms in glaucoma and the need for reliable molecular biomarkers, this integrative strategy facilitates candidate gene prioritization and provides a basis for hypothesis generation in glaucoma research.

## 2. Materials and Methods

### 2.1. Data Download

Bulk transcriptomic datasets were downloaded from the Gene Expression Omnibus (GEO) database using the GEOquery R package (version 2.78.0). Expression matrices and corresponding sample metadata were retrieved for downstream analysis.

Log2 transformation was applied when required based on quantile distribution inspection to ensure approximate normality. Probes were mapped to gene symbols using platform annotation (GPL) files. Probes without valid gene annotations were removed. When multiple probes corresponded to the same gene symbol, the probe with the highest mean expression across samples was retained.

Sample grouping (POAG vs. Control) was defined according to metadata fields in the pData table and standardized prior to integration with the expression matrix. Samples and expression profiles were matched to ensure consistency before downstream analysis.

### 2.2. Differential Analysis

The Limma package (version 3.66.0) was used to identify differentially expressed genes (DEGs) between glaucoma and control samples, with empirical Bayes moderation of standard errors. DEGs were defined using a significance threshold of *p* < 0.05 and |logFC| > 1.

### 2.3. Single-Cell RNA Sequencing Data Processing

Single-cell RNA sequencing data were processed using the Seurat R packageversion (5.4.0). Raw count matrices were imported using the CreateSeuratObject function with filtering thresholds of min.cells = 3 and min.features = 200. The H9RimS1 sample was excluded before quality control procedures.

For each cell, the following quality control metrics were calculated: total UMI counts (nCount_RNA), number of detected genes (nFeature_RNA), percentage of mitochondrial genes (percent. mt), and percentage of ribosomal genes (percent.ribo). Cells deviating more than three median absolute deviations (MADs) from the median for any metric were removed. Cells with abnormally high UMI counts or gene numbers were excluded to reduce potential doublets. Cells with elevated mitochondrial or ribosomal gene percentages were removed to eliminate low-quality or stressed cells.

Data normalization was performed using the LogNormalize method, scaling each cell to a total of 10,000 counts followed by log transformation. Cell cycle scores (S.Score and G2M. Score) were calculated using the CellCycleScoring function. Highly variable genes were identified using FindVariableFeatures (method = “vst”, nfeatures = 2000). Prior to dimensional reduction, data were scaled using the ScaleData function, regressing out variation due to mitochondrial percentage (percent.mt), ribosomal percentage (percent.ribo), and cell cycle scores (S.Score and G2M.Score). Principal component analysis was performed using RunPCA (npcs = 50). The first 20 principal components were used for clustering. For multi-sample integration, batch effects were corrected using the Harmony algorithm. Uniform Manifold Approximation and Projection (UMAP) visualization and clustering were performed using the Harmony-corrected embeddings. Cell-type annotation was performed based on canonical marker genes reported in the literature, the CellMarker database, and the results of automated annotation using the SingleR package (version 2.12.0). Final cluster identities were assigned based on concordant results across annotation approaches.

### 2.4. Functional Enrichment Analysis

To explore the biological functions and pathways associated with DEGs, gene ontology (GO) and Kyoto Encyclopedia of Genes and Genomes (KEGG) enrichment analyses were conducted using the clusterProfiler R package (version 4.18.4). Enrichment results were considered statistically significant at *p* < 0.05 after multiple testing correction. Enriched biological processes, cellular components, molecular functions, and pathways were visualized to facilitate biological interpretation.

### 2.5. Weighted Gene Co-Expression Network Analysis (WGCNA)

Weighted gene co-expression network analysis (WGCNA), using the WGCNA R package (version 1.73), was performed to identify co-expressed gene modules associated with POAG. Prior to network construction, low-variance genes were filtered to reduce noise. Sample clustering was performed to identify potential outliers, and one outlier sample was removed based on a cut height of 100. The final dataset included 35 samples for WGCNA. The soft-thresholding power was determined using the pickSoftThreshold function with powerVector = 1:20 and RsquaredCut = 0.85. The selected soft-thresholding power was 9. The network was constructed using networkType = “unsigned” and TOMType = “unsigned”. Gene modules were identified using the dynamic tree cut algorithm with deepSplit = 2 and minModuleSize = 30. Module merging was performed using mergeCutHeight = 0.25. Genes were clustered into modules using hierarchical clustering, and module eigengenes were calculated. The correlation between module eigengenes and disease status was assessed to identify disease-associated modules. Lactylation-related gene sets were integrated to prioritize modules potentially linked to metabolic regulation.

### 2.6. Machine Learning for Prediction Model

To identify candidate biomarkers with potential diagnostic value, multiple machine learning algorithms were evaluated using the caret package (version 7.0-1). The input features consisted of normalized gene expression values of candidate genes derived from the intersection of differentially expressed genes (DEGs) and the lactylation-associated WGCNA module. Each sample was represented as a feature vector containing the expression values of these genes. The output of each model was a predicted probability score indicating the likelihood that a sample belonged to the POAG group. Probability scores were converted into binary class labels using a threshold of 0.5 for performance evaluation. Model performance was assessed using accuracy, sensitivity, specificity and the area under the receiver operating characteristic curve (AUC). To mitigate overfitting risks associated with the moderate sample size, we restricted feature dimensionality to biologically relevant candidate genes and applied internal cross-validation during model training. Model generalizability was further assessed using an independent external dataset (GSE9944).

### 2.7. Immune Infiltration Analysis

The CIBERSORT algorithm was applied to the GSE27276 bulk transcriptomic dataset to estimate the relative proportions of immune cell types within POAG and control samples. Differences in immune cell infiltration between groups were compared, and Spearman correlation analysis was performed to assess associations between the expression levels of potential diagnostic model genes and immune cell populations. Immune infiltration analysis was conducted independently from the machine learning pipeline and was not involved in feature selection or model construction.

### 2.8. GSVA and GSEA

Gene set variation analysis (GSVA) was performed using the GSVA package (version 2.4.7) to evaluate pathway activity changes between glaucoma and control samples at the individual sample level. This method generates an enrichment score for each sample based on coordinated expression of the predefined gene set. Specifically, a lactylation-related gene set was defined, and the ssGSEA method within the GSVA package was used to calculate a lactylation score for each sample as a single-sample enrichment score. This score was then incorporated as a continuous trait in WGCNA for module–trait correlation analysis. In parallel, gene set enrichment analysis (GSEA) was conducted to identify significantly enriched biological pathways in association with disease status. Curated gene sets were obtained from the Molecular Signatures Database (MSigDB), and pathways with FDR < 0.25 were considered significant.

### 2.9. Drug–Gene Interaction Prediction

Potential drug–gene interactions for the identified candidate genes were predicted using the Drug–Gene Interaction Database (DGIDB). Interactions supported by curated databases or experimental evidence were retained, providing preliminary insights into potential therapeutic targets for glaucoma.

### 2.10. Quality Control and Data Standardization

Quality control and downstream analysis of single-cell RNA sequencing data were performed using the Seurat package (version 5.4.0). Cells with low gene counts, excessive mitochondrial gene expression, or abnormal library sizes were excluded. Data were normalized and then subjected to principal component analysis (PCA). Batch effects across samples were corrected using the Harmony algorithm. The processed single-cell data were subsequently used to examine the cell type-specific expression patterns of candidate genes identified from bulk analyses.

## 3. Results

### 3.1. Global Transcriptomic Alterations in POAG

**Figure 1:**
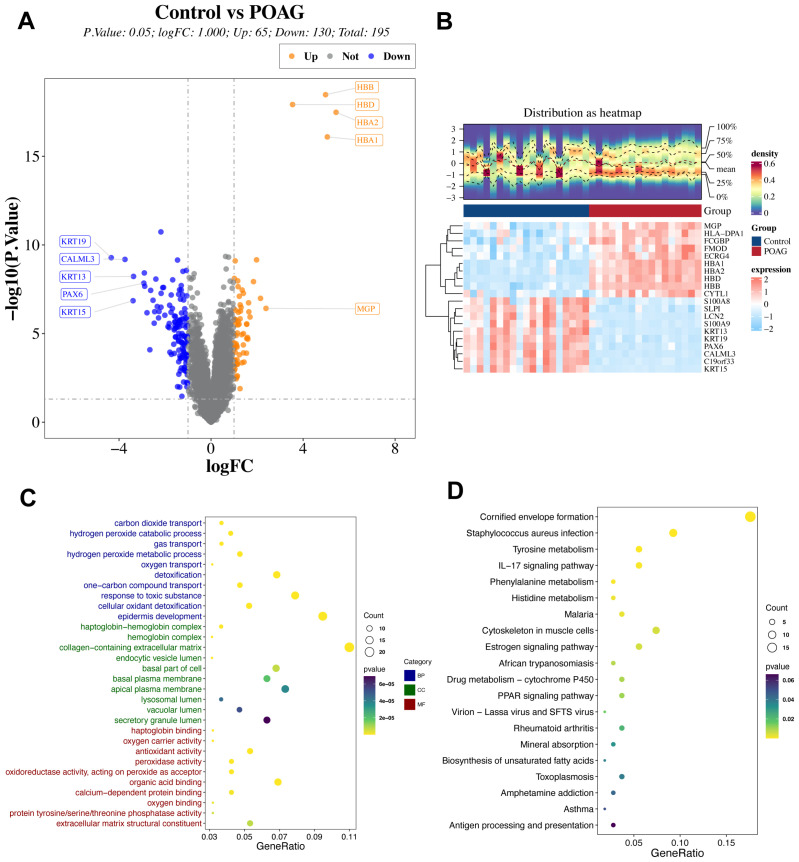
Differential gene expression analysis of Control vs. Primary open-angle glaucoma (POAG). (**A**) Volcano plot showing the differential gene expression between the control and POAG groups. The x-axis represents the log2 fold change (logFC), and the y-axis represents the -log10 of the *p*-Value. Genes significantly upregulated in POAG are shown in orange, while genes downregulated in POAG are depicted in blue. The total number of differentially expressed genes is 195, with 65 upregulated and 130 downregulated. (**B**) Heatmap displaying the distribution of expression levels for the top differentially expressed genes across the control and POAG groups. The heatmap shows gene expression in the two groups, with higher expression levels represented in red and lower levels in blue. A hierarchical clustering approach was used to group the genes based on expression patterns. (**C**) Gene ontology (GO) enrichment analysis of differentially expressed genes. The plot shows the top enriched biological processes (BP), cellular components (CC), and molecular functions (MF) associated with the DEGs. The size of the dots represents the count of genes in each category, while the color indicates the significance of the enrichment (*p*-Value). (**D**) KEGG pathway enrichment analysis of differentially expressed genes. The plot highlights the top enriched signaling pathways and processes. Pathways related to immune response, metabolism, and cell structure are prominently represented, such as the IL-17 signaling pathway, tyrosine metabolism, and corneous envelope formation.

Differential expression analysis was performed between control and primary open-angle glaucoma (POAG) samples to investigate global transcriptomic alterations. A total of 195 differentially expressed genes (DEGs) were identified, comprising 65 upregulated and 130 downregulated genes in POAG (Figure 1A). Hierarchical clustering based on these DEGs separated POAG samples from controls (Figure 1B), demonstrating substantial transcriptional differences between the groups.

Functional enrichment analysis revealed that DEGs were predominantly enriched in biological processes related to oxidative stress response and extracellular matrix organization (Figure 1C). These findings, along with previous studies, indicate that oxidative stress and structural alterations of the trabecular meshwork are central features of glaucomatous pathology [4,15]. KEGG pathway analysis further identified enrichment in immune- and metabolism-related pathways (Figure 1D). This finding supports that POAG is associated with complex molecular and metabolic dysregulation rather than isolated gene-level changes [2].

### 3.2. Identification and Validation of Lactylation-Associated Gene Modules

To investigate whether protein lactylation might contribute to POAG pathogenesis at the transcriptomic level, we performed WGCNA. Lactylation-related genes were first obtained from public databases, and lactylation scores were calculated for each sample via ssGSEA (using the GSVA package) based on the expression of these genes. Based on transcriptomic expression profiles, we constructed a scale-free co-expression network from the transcriptomic data, and genes were clustered into distinct modules.

**Figure 2:**
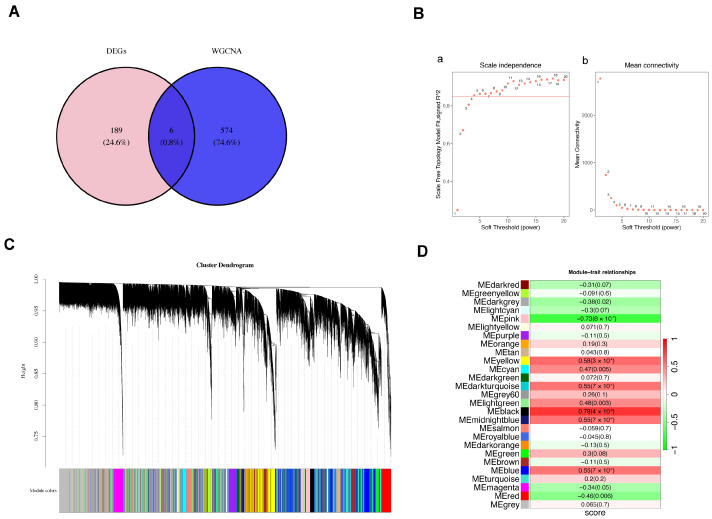
Weighted Gene Co-expression Network Analysis (WGCNA) and module–trait relationships. (**A**) Venn diagram showing the overlap between the differentially expressed genes (DEGs) and the modules identified with WGCNA. Of the 195 DEGs, 6 overlap with the WGCNA modules, and 189 are not part of the WGCNA network. (**B**) Selection of the soft threshold power for WGCNA. Panel (**a**) shows the scale-free topology model fit index (R^2^) as a function of the soft-thresholding (power), indicating the optimal threshold for network construction. Panel (**b**) displays the mean connectivity across different soft threshold powers; (**C**) Cluster dendrogram showing the hierarchical clustering of genes based on their expression patterns. Genes are clustered into distinct modules, and the colors below the dendrogram represent the assigned module colors. (**D**) Module–trait relationship analysis. This heatmap shows the correlation between each module and clinical traits. The numbers on the right represent the correlation coefficients, and the colors indicate the strength and direction of the correlation. Significant positive correlations are marked in green, while negative correlations are shown in red.

Among all identified modules, the black module showed the strongest positive correlation with both lactylation scores and the POAG phenotype (Figure 2B–D). To further narrow down the range of key genes, we conducted cross-analysis of DEGs and genes within the black module. Six overlapping genes were identified and selected as lactylation-associated candidate genes for subsequent analyses (Figure 2A). This network-based approach has been widely applied to dissect complex diseases, helping to shift the focus from individual genes to a coordinated gene network linked to lactylation [16]. The discovery of a lactylation-associated gene module—consistent with the emerging role of lactylation as a key regulatory mechanism [12]—provides a potential framework for subsequent analyses.

To assess the diagnostic potential of lactylation-associated candidate genes, multiple machine learning classifiers were trained and compared. Comparative performance across algorithms is summarized in Supplementary Figure S2. Among the evaluated models, Random Forest and Gradient Boosting Machine demonstrated superior predictive performance on both the training and external validation datasets. Feature importance analysis consistently identified *S100A2* and *S100A14* as the most informative predictors distinguishing POAG from controls.

These findings support the diagnostic relevance of *S100A2* and *S100A14* and provide the basis for subsequent biological analyses [14].

### 3.3. Immune-Related Compositional Changes in POAG

**Figure 3:**
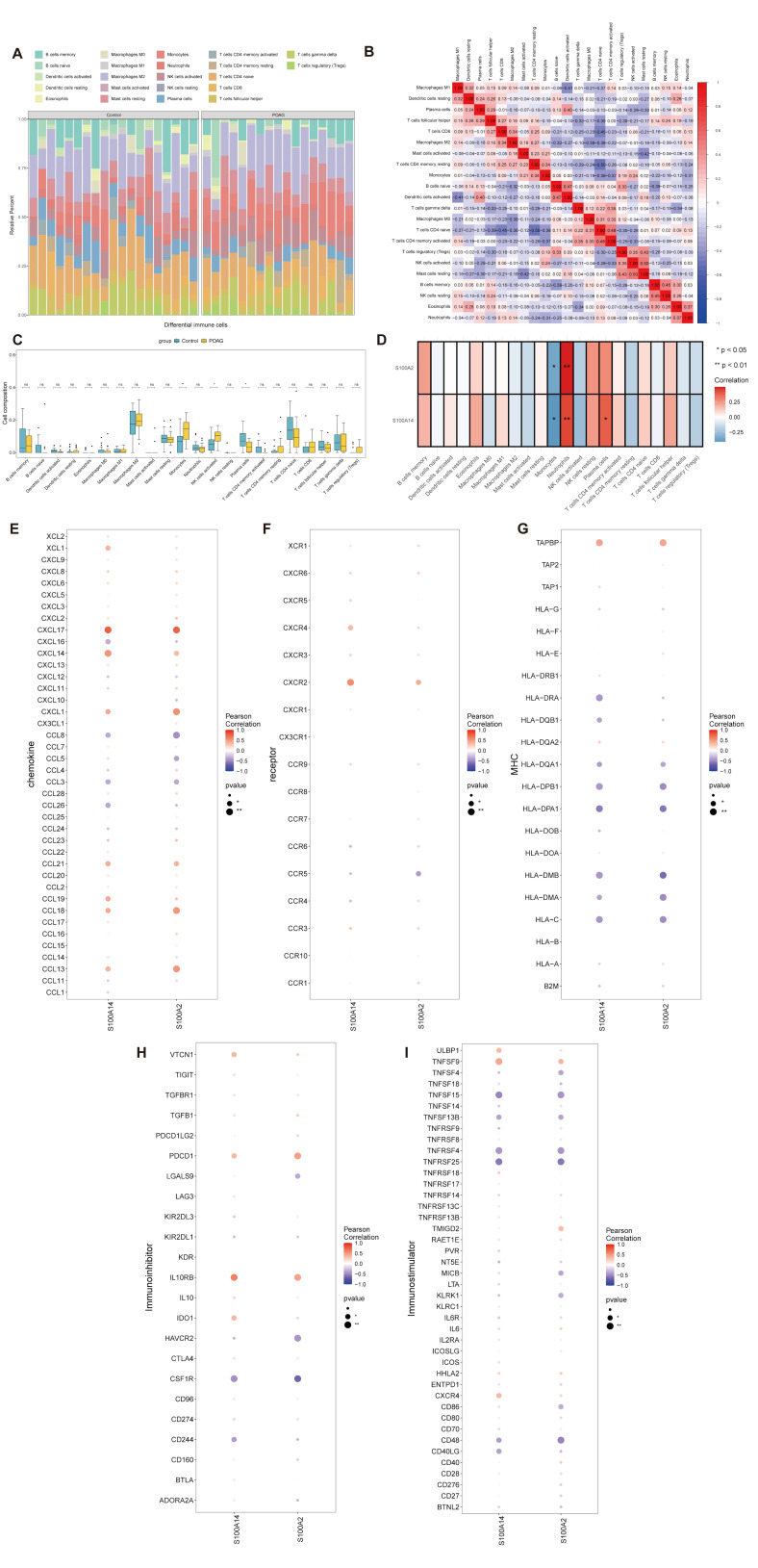
Immune infiltration analysis and immune-related correlations of *S100A2* and *S100A14* in POAG. (**A**) Stacked bar plot showing the relative proportions of immune cell types in control and POAG samples estimated using the CIBERSORT algorithm. Each color represents a distinct immune cell population. This panel is presented for visualization of compositional patterns. (**B**) Correlation matrix among immune cell populations. Pearson correlation coefficients are displayed, with red indicating positive correlations and blue indicating negative correlations. (**C**) Comparison of immune cell fractions between control and POAG groups. Statistical differences were evaluated using the Wilcoxon rank-sum test, and *p*-values were adjusted using the Benjamini–Hochberg method. Significance levels are indicated as * *p* < 0.05; ** *p* < 0.01; ns, not significant. (**D**) Correlation analysis between *S100A2* and *S100A14* expression levels and immune cell proportions. Pearson correlation coefficients are shown, with color intensity reflecting the direction and strength of the association. Statistical significance is indicated as * *p* < 0.05; ** *p* < 0.01. (**E**–**G**) Correlation analyses between *S100A2*/*S100A14* expression and immune-related gene signatures in POAG samples, including chemokines (**E**), immune receptors (**F**), and HLA-related genes (**G**). Dot color represents Pearson correlation coefficient, and dot size reflects statistical significance. Only correlations with *p* < 0.05 are highlighted. Correlations that did not reach statistical significance should be interpreted as exploratory. (**H**) Correlation between immune inhibitors (e.g., *PDCD1* and *CTLA4*) and immune markers in the POAG group. The plot highlights significant negative and positive correlations with color intensity. (**I**) Correlation between immune stimulators (e.g., *CD80* and *CD44*) and immune markers in the POAG group. The plot indicates significant correlations, with color gradients representing the correlation strength.

Considering the important role of immune dysregulation in glaucoma, we next investigated immune microenvironment remodeling in POAG [6,7,15]. CIBERSORT-based deconvolution suggested altered immune-cell profiles in POAG, and groupwise comparison indicated significant differences in a subset of immune cell fractions, including monocytes, neutrophils, plasma cells, and signatures of selected activated immune cells, relative to controls (Figure 3A–C).

Correlation analysis further demonstrated that *S100A2* and *S100A14* were associated with the infiltration levels of immune cells, particularly monocyte- and neutrophil-related fractions. In addition, significant correlations were observed between model genes and chemokines, immune receptors, major histocompatibility complex (MHC) molecules, immune inhibitors, and immune stimulators (Figure 3D–I), suggesting that these genes may participate in immune-related transcriptional regulation and inflammatory signaling during POAG progression. Thus, our data link *S100A2* and *S100A14* to selected immune-related alterations in POAG, supporting the broader concept that immune dysregulation contributes to glaucomatous damage [7,15].

### 3.4. Pathway and Regulatory Network Analysis of Model Genes

**Figure 4:**
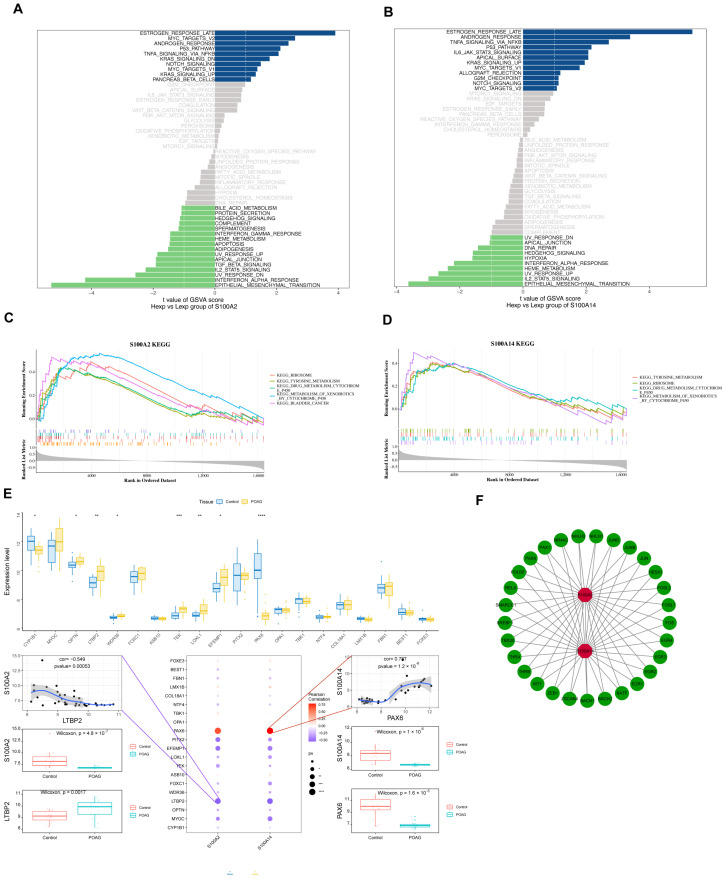
Pathway enrichment and regulatory network analysis of the model genes. (**A**,**B**) Gene set variation analysis (GSVA) showing differential pathway activity between high- and low-expression groups of *S100A2* (**A**) and *S100A14* (**B**). Pathways are ranked according to GSVA score differences, highlighting immune-, metabolic-, and hormone-related signaling signatures. (**C**) Gene set enrichment analysis (GSEA) for the *S100A2* gene. This plot shows the running enrichment score for several KEGG pathways related to *S100A2.* The pathways include KEGG Ribosome, Tyrosine Metabolism, Drug Metabolism by Cytochrome P450, Metabolism of Xenobiotics, and Bladder Cancer. (**D**) Gene set enrichment analysis (GSEA) for the *S100A14* gene. This plot shows the running enrichment score for several KEGG pathways related to *S100A14*, including Tyrosine Metabolism, Ribosome, and Cytochrome P450-mediated Drug Metabolism. (**E**) Box plot showing the expression levels of key genes in control and POAG tissues. The expression of genes such as *LTBP2* and *PAX6* is significantly altered in POAG. Below the box plot, the correlation between LTBP2 and S100A2, as well as between *PAX6* and *S100A2*, is shown, along with the statistical significance for each correlation. (**F**) Network showing the interactions between transcription factors and genes. *S100A2* and *S100A14* are central in the network, with numerous transcription factors (e.g., *PAX9*, *RELA*, *JUN)* connected to them. The edges represent regulatory relationships between the nodes (genes). Statistical significance is indicated as follows: ns, not significant; * *p* < 0.05, ** *p* < 0.01, *** *p* < 0.001, **** *p* < 0.0001.

Following the observation that *S100A2* and *S100A14* were associated with immune cell infiltration and immune-related signatures, we next sought to determine whether these model genes were embedded within broader functional pathways and regulatory programs relevant to POAG. To this end, pathway enrichment analyses were performed to further elucidate the biological functions of the model genes. GSVA revealed that *S100A2* and *S100A14* were mainly enriched in hormone response-related, immune-related, and metabolism-related pathways (Figure 4A,B).

Consistently, GSEA demonstrated significant enrichment of ribosome, tyrosine metabolism, and cytochrome P450-associated pathways (Figure 4C,D), indicating coordinated pathway-level alterations rather than isolated gene effects. Beyond pathway association, we also found that *S100A2* and *S100A14* correlated significantly with known glaucoma-related genes. To explore their upstream regulation, we performed transcription factor motif analysis, which identified potential regulators and outlined a putative transcriptional network involved in POAG pathogenesis (Figure 4E,F).

Collectively, these pathway- and regulation-oriented analyses suggest that *S100A2* and *S100A14* are embedded within coordinated functional programs involving metabolic and immune-associated processes in POAG. However, these results are derived from bulk transcriptomic profiles and therefore do not resolve the cellular sources or cell type-specific contexts underlying these associations. To address this limitation and to determine how the model genes are distributed across distinct cellular populations, we next performed single-cell RNA sequencing based analyses.

### 3.5. Single-Cell Validation and Drug Prediction

Rigorous quality control was performed prior to downstream single-cell analyses. Cells were filtered based on gene number, unique molecular identifier (UMI) counts, and mitochondrial and ribosomal gene proportions, resulting in 20,312 high-quality cells retained for subsequent analysis (Supplementary Figure S1A,B). After normalization, dimensionality reduction using principal component analysis (PCA) and batch effect correction were applied, enabling robust integration across samples (Supplementary Figure S1C–F).

**Figure 5:**
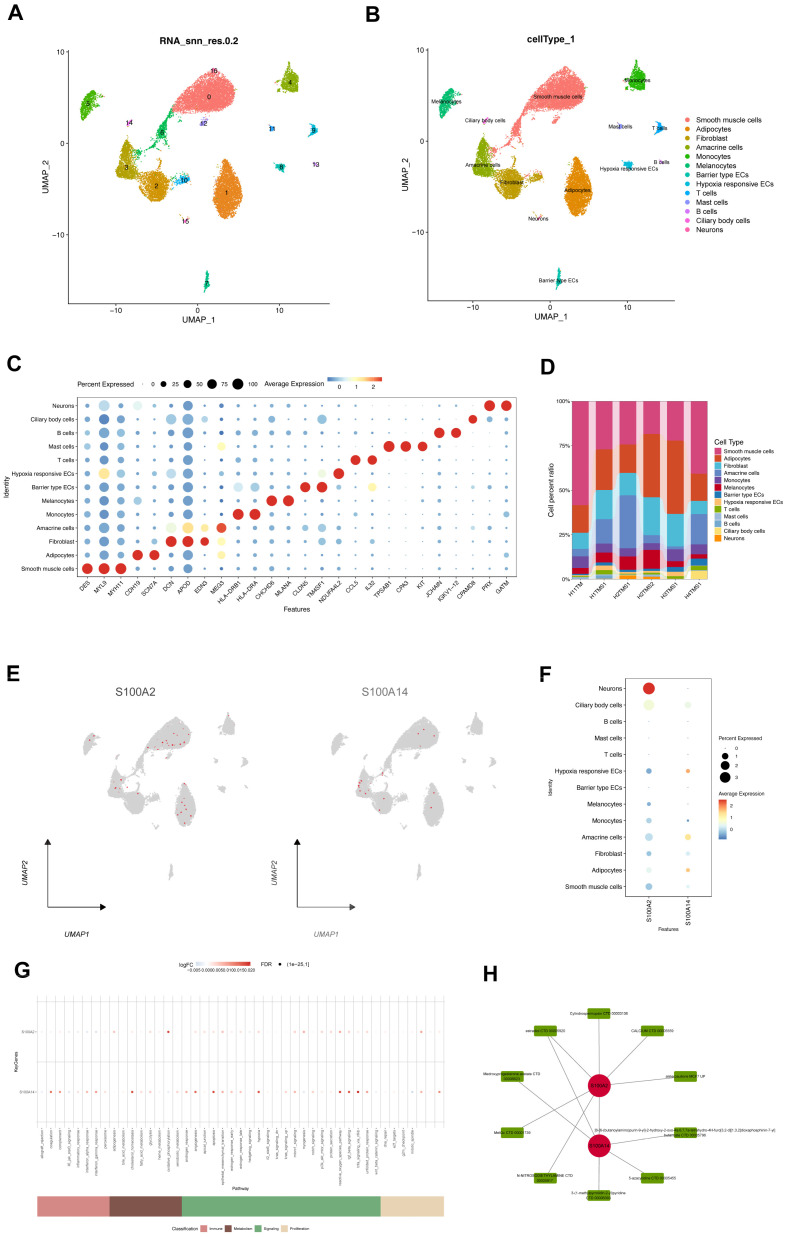
Single-cell validation and drug prediction. (**A**) Uniform Manifold Approximation and Projection (UMAP) plot showing the clustering of cells based on RNA expression profiles. Each cluster is labeled with a unique number, representing distinct cell populations identified in the data. The corresponding biological cell-type annotations are shown in panel (**B**). (**B**) UMAP plot showing cell type annotation. Cells are colored according to their identified cell type, including smooth muscle cells (red), adipocytes (orange), fibroblasts (green), amacrine cells (light green), monocytes (light blue), melanocytes (purple), barrier-type endothelial cells (blue), hypoxia-responsive endothelial cells (dark blue), T cells (yellow), mast cells (pink), B cells (light red), ciliary body cells (gray), and neurons (dark green). (**C**) Dot plot showing the expression of key marker genes for each identified cell type. The size of each dot represents the percentage of cells expressing a specific gene, while the color intensity represents the average expression level of that gene in the corresponding cell type. (**D**) Stacked bar chart showing the cell type distribution across different sample groups. The proportions of each cell type are displayed for the different groups, showing the variation in cell type composition between the groups. (**E**) UMAP plot showing the expression distribution of *S100A2* and *S100A14* across all cells. The color gradient represents the expression level, with darker red indicating higher expression. (**F**) Dot plot showing the expression of *S100A2* and *S100A14* across different cell types. The size of the dots represents the percentage of cells expressing the gene, and the color intensity indicates the average expression level in each cell type. (**G**) Pathway enrichment analysis for *S100A2* and *S100A14*. This plot shows the top enriched pathways for both genes, with pathways categorized under immune response, metabolism, signaling, and proliferation. The color gradient represents the log2 fold change, and the size of the dots corresponds to the significance of the enrichment (FDR). (**H**) Drug interaction network of *S100A2* and *S100A14*. The network displays potential drug compounds (green nodes) that interact with these genes, including drug classes and related targets, suggesting potential therapeutic applications.

Unsupervised clustering followed by UMAP visualization identified multiple distinct cell populations, which were annotated using established marker genes (Figure 5A–D). Both *S100A2* and *S100A14* showed sparse but non-uniform expression across cell populations, with relative enrichment in selected cell types (Figure 5E,F), highlighting cellular context that cannot be resolved by bulk transcriptomic analyses alone.

To further characterize functional states at the single-cell level, AUCell scoring was applied to evaluate pathway-associated gene set activity. This analysis revealed differential immune- and metabolism-related activity across cell clusters (Figure 5G), providing complementary functional context for the bulk-derived model genes. Similar single-cell strategies have previously highlighted cellular heterogeneity in glaucomatous tissues [11].

Notably, single-cell analyses in this study were primarily designed to serve as cellular-context validation of bulk transcriptomic findings rather than to reconstruct dynamic cellular trajectories or intercellular communication networks. Given that the available dataset represents a static transcriptional snapshot and that trabecular meshwork tissue is non-hematopoietic in nature, trajectory inference and cell-cell communication analyses were not pursued. Accordingly, the single-cell results are interpreted as reflecting cell type-associated transcriptional states rather than directional or causal cellular processes. In addition, drug-gene interaction analysis based on DGIDB identified candidate compounds potentially targeting *S100A2* and *S100A14* (Figure 5H), providing a preliminary reference for future experimental and clinical studies.

## 4. Discussion

Collectively, this study establishes an integrative framework that links metabolic and immune-related transcriptional alterations to candidate diagnostic biomarkers in POAG. In this multi-omics framework, we conducted an integrative analysis combining bulk transcriptomics, single-cell RNA sequencing, immune deconvolution, and machine learning to investigate molecular alterations associated with POAG. By basing our analysis on lactylation-associated gene modules and validating findings across multiple analytical layers, we identified *S100A2* and *S100A14* as candidate diagnostic biomarkers and explored their potential biological context.

To understand the potential biological background of these markers, we conducted a systematic analysis of the global transcriptome alterations of POAG. Global transcriptomic analysis revealed widespread gene expression changes in POAG, consistent with previous reports demonstrating extensive molecular remodeling in glaucomatous trabecular meshwork and retinal tissues [9]. Functional enrichment analyses highlighted oxidative stress responses and extracellular matrix organization, both of which have been recognized as central pathological features of glaucoma [4,5].

By incorporating lactylation-associated genes into a weighted gene co-expression network framework, our study extends prior transcriptomic analyses by linking metabolic reprogramming to disease-relevant gene modules. Protein lactylation has emerged as a key mechanism connecting altered cellular metabolism to transcriptional regulation under pathological conditions [14]. Although the present study infers lactylation involvement at the transcriptomic level, the identified module provides a transcriptomic context for exploring metabolic regulation in POAG.

We further utilize integrated machine learning strategies to prioritize core candidate genes with potential diagnostic value from these modules. A major strength of this work is the use of an ensemble machine learning strategy to prioritize candidate biomarkers. Ensemble approaches have been shown to outperform individual algorithms in glaucoma classification and biomarker discovery tasks [17]. Using this framework, *S100A2* and *S100A14* were consistently identified as the most informative predictors.

Members of the S100 protein family are known to participate in calcium signaling, inflammatory responses, and cellular stress pathways [18]. Previous studies have reported altered expression of S100 family proteins in various ocular and neuroinflammatory conditions, supporting the potential relevance of *S100A2* and *S100A14* in glaucoma-associated molecular processes.

Furthermore, immune infiltration analysis demonstrated altered immune-related compositional patterns in POAG, with model genes showing consistent associations with immune cells and immune regulatory molecules. Increasing evidence supports a role for immune dysregulation and neuroinflammation in glaucomatous optic neuropathy [6,7]. Importantly, these observations should be interpreted as associative rather than causal. The limited statistical significance observed in several correlation analyses may reflect the moderate sample size and the inherent variability associated with bulk transcriptomic-based immune deconvolution methods.

Moreover, given the non-hematopoietic nature of trabecular meshwork tissue, immune deconvolution-based estimates reflect inferred immune-related transcriptional signatures rather than direct measurements of immune cell infiltration and should therefore be interpreted with appropriate caution. Nevertheless, the consistency between immune-associated patterns and pathway enrichment results supports the relevance of immune-related processes in POAG, as previously reported in experimental and clinical studies [19].

To further examine the accuracy of our research at the cellular level, we introduced single-cell RNA sequencing data for in-depth analysis. The incorporation of single-cell RNA sequencing data represents another key strength of this study. Single-cell analyses showed that *S100A2* and *S100A14* exhibited sparse but non-uniform expression across cell populations, underscoring the importance of cellular context in interpreting POAG-related transcriptomic findings. Prior single-cell studies have demonstrated substantial cellular heterogeneity in ocular tissues that cannot be resolved using bulk transcriptomic approaches alone [11].

By validating bulk transcriptomic findings at single-cell resolution, our study mitigates the confounding effect of cell composition changes and supports the biological relevance of the identified biomarkers.

As a preliminary exploratory step, the identification of *S100A2* and *S100A14* as candidate diagnostic biomarkers may inform future assay development, pending clinical validation for early disease detection or patient stratification. Moreover, drug–gene interaction analysis provides a preliminary framework for therapeutic exploration, aligning with recent efforts to repurpose existing drugs for glaucoma treatment [20].

However, multiple glaucoma-associated biomarkers have been reported previously, but routine POAG diagnosis still relies primarily on established clinical and imaging parameters. This is largely because most molecular biomarkers remain at the candidate stage and have not yet undergone sufficient prospective validation, assay standardization, or demonstration of incremental clinical value beyond existing diagnostic approaches.

The feasibility of the analytical framework is supported by the use of publicly available datasets and well-established computational tools, suggesting that this strategy can be readily applied to other ocular diseases.

Although the above analytical framework shows good application prospects, there are still some limitations of this study that deserve attention. First, it relies primarily on retrospective public datasets with a moderate sample size. Although an independent external cohort was used for validation, the machine learning analysis should be interpreted as an exploratory analysis for candidate biomarker prioritization rather than as a formal clinical validation. Larger prospective clinical cohorts are required to further assess generalizability and real-world utility. Second, lactylation-related associations were inferred computationally and were not experimentally validated at the protein modification level. Third, while single-cell analyses provided valuable validation, current datasets remain limited in sample size and tissue diversity, which may restrict resolution of disease heterogeneity.

Future studies incorporating experimental validation of lactylation events, functional assays in relevant ocular cell types, and longitudinal clinical cohorts will be essential to further elucidate the mechanistic and clinical relevance of *S100A2* and *S100A14* in POAG.

## 5. Conclusions

In conclusion, this study presents an integrative analytical framework linking transcriptomic remodeling, metabolic regulation, immune-related alterations, and machine learning-based classification analysis in POAG. By prioritizing S100A2 and S100A14 as lactylation-associated biomarkers, our findings provide new insights into the molecular heterogeneity of POAG and provide a basis for future mechanistic and validation studies.

## Data Availability

The datasets analyzed in this study are publicly available in the Gene Expression Omnibus (GEO) database under accession numbers GSE148371, GSE27276, and GSE9944. All processed data supporting the findings of this study are included within the article and its Supplementary Materials.

## Supplementary Materials

The following supporting information can be downloaded at: https://www.mdpi.com/article/10.3390/genes17040403/s1, Figure S1: Quality control and principal component analysis of single-Cell RNA-seq data; Figure S2: Machine learning model performance evaluation.

## Abbreviations

The following abbreviations are used in this manuscript:

POAG	Primary open-angle glaucoma
scRNA-seq	Single-cell RNA sequencing
WGCNA	Weighted gene co-expression network analysis
GEO	Gene Expression Omnibus
IOP	Intraocular pressure
KEGG	Kyoto Encyclopedia of Genes and Genomes
GSVA	Gene set variation analysis
GSEA	Gene set enrichment analysis
MSigDB	Molecular Signatures Database
DGIDB	Drug–Gene Interaction Database
PCA	Principal component analysis
DEGs	Differentially expressed genes
NCBI	National Center for Biotechnology Information
NIH	National Institutes of Health

## References

[1] Weinreb R.N., Leung C.K.S., Crowston J.G., Medeiros F.A., Friedman D.S., Wiggs J.L., Martin K.R. (2016). Primary Open-Angle Glaucoma. Nat. Rev. Dis. Primer.

[2] Weinreb R.N., Aung T., Medeiros F.A. (2014). The Pathophysiology and Treatment of Glaucoma: A Review. JAMA.

[3] Tham Y.-C., Li X., Wong T.Y., Quigley H.A., Aung T., Cheng C.-Y. (2014). Global Prevalence of Glaucoma and Projections of Glaucoma Burden through 2040: A Systematic Review and Meta-Analysis. Ophthalmology.

[4] Rohen J.W., Witmer R. (1972). Electrn Microscopic Studies on the Trabecular Meshwork in Glaucoma Simplex. Albrecht von Graefes Arch. Klin. Ophthalmol..

[5] Tezel G. (2006). Oxidative Stress in Glaucomatous Neurodegeneration: Mechanisms and Consequences. Prog. Retin. Eye Res..

[6] Williams P.A., Marsh-Armstrong N., Howell G.R., The Lasker/IRRF Initiative on Astrocytes and Glaucomatous Neurodegeneration Participants (2017). Neuroinflammation in Glaucoma: A New Opportunity. Exp. Eye Res..

[7] Howell G.R., Soto I., Zhu X., Ryan M., Macalinao D.G., Sousa G.L., Caddle L.B., MacNicoll K.H., Barbay J.M., Porciatti V. (2012). Radiation Treatment Inhibits Monocyte Entry into the Optic Nerve Head and Prevents Neuronal Damage in a Mouse Model of Glaucoma. J. Clin. Investig..

[8] Greatbatch C.J., Lu Q., Hung S., Barnett A.J., Wing K., Liang H., Han X., Zhou T., Siggs O.M., Mackey D.A. (2024). High Throughput Functional Profiling of Genes at Intraocular Pressure Loci Reveals Distinct Networks for Glaucoma. Hum. Mol. Genet..

[9] Liton P.B., Luna C., Challa P., Epstein D.L., Gonzalez P. (2006). Genome-Wide Expression Profile of Human Trabecular Meshwork Cultured Cells, Nonglaucomatous and Primary Open Angle Glaucoma Tissue. Mol. Vis..

[10] Stuart T., Butler A., Hoffman P., Hafemeister C., Papalexi E., Mauck W.M., Hao Y., Stoeckius M., Smibert P., Satija R. (2019). Comprehensive Integration of Single-Cell Data. Cell.

[11] van Zyl T., Yan W., McAdams A., Peng Y.-R., Shekhar K., Regev A., Juric D., Sanes J.R. (2020). Cell Atlas of Aqueous Humor Outflow Pathways in Eyes of Humans and Four Model Species Provides Insight into Glaucoma Pathogenesis. Proc. Natl. Acad. Sci. USA.

[12] Zhang D., Tang Z., Huang H., Zhou G., Cui C., Weng Y., Liu W., Kim S., Lee S., Perez-Neut M. (2019). Metabolic Regulation of Gene Expression by Histone Lactylation. Nature.

[13] Chen J., Huang Z., Chen Y., Tian H., Chai P., Shen Y., Yao Y., Xu S., Ge S., Jia R. (2025). Lactate and Lactylation in Cancer. Signal Transduct. Target. Ther..

[14] Li Z., He Y., Keel S., Meng W., Chang R.T., He M. (2018). Efficacy of a Deep Learning System for Detecting Glaucomatous Optic Neuropathy Based on Color Fundus Photographs. Ophthalmology.

[15] Tezel G. (2011). The Immune Response in Glaucoma: A Perspective on the Roles of Oxidative Stress. Exp. Eye Res..

[16] Langfelder P., Horvath S. (2008). WGCNA: An R Package for Weighted Correlation Network Analysis. BMC Bioinform..

[17] Wang X., Chen H., Ran A.-R., Luo L., Chan P.P., Tham C.C., Chang R.T., Mannil S.S., Cheung C.Y., Heng P.-A. (2020). Towards Multi-Center Glaucoma OCT Image Screening with Semi-Supervised Joint Structure and Function Multi-Task Learning. Med. Image Anal..

[18] Donato R., Cannon B.R., Sorci G., Riuzzi F., Hsu K., Weber D.J., Geczy C.L. (2013). Functions of S100 Proteins. Curr. Mol. Med..

[19] Almasieh M., Wilson A.M., Morquette B., Cueva Vargas J.L., Di Polo A. (2012). The Molecular Basis of Retinal Ganglion Cell Death in Glaucoma. Prog. Retin. Eye Res..

[20] Quigley H.A., Broman A.T. (2006). The Number of People with Glaucoma Worldwide in 2010 and 2020. Br. J. Ophthalmol..

