# Supplementary material for: Lactylation-Associated Immune Metabolic Reprogramming Identifies S100A2 and S100A14 as Candidate Diagnostic Biomarkers in Primary Open-Angle Glaucoma: An Integrated Bulk and Single-Cell Transcriptomic Analysis
Source: Genes (Basel). 2026 Mar 31;17(4):403. doi: 10.3390/genes17040403 (PMC13115881; doi:10.3390/genes17040403)
Supplement: Supplementary file 1 [file genes-17-00403-s001.zip › genes-4175394-supplementary.pdf]

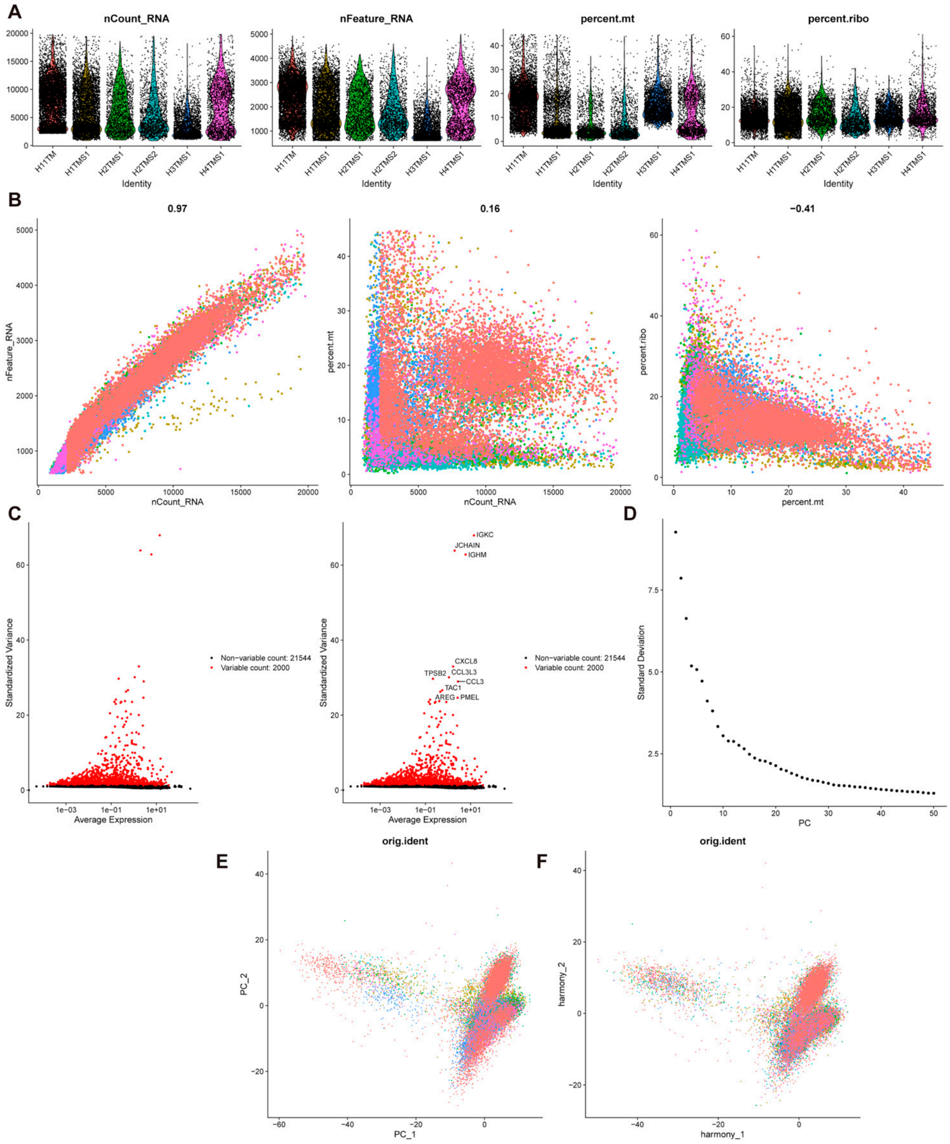

Supplementary Figure S1. Quality control and principal component analysis of single-Cell RNA-seq data. (**A**) Distribution of the number of detected genes (nFeature_RNA), total UMI counts (nCount_RNA), mitochondrial gene percentage (percent.mt), and ribosomal gene percentage (percent.ribo) across samples prior to filtering. (**B**) Scatter plots illustrating relationships between nCount_RNA and nFeature_RNA, nCount_RNA and percent.mt, and percent.mt and percent.ribo, used to assess data quality and identify potential low-quality cells. (**C**) Scatter plot showing the variance in expression versus average expression for all genes. Variable genes (highlighted in red) are shown separately from non-variable genes (black). (**D**) Scree plot showing the explained variance of the principal components (PCs) of the dataset. The plot illustrates how much variance is

explained by each PC. (**E**) PCA plot showing the first two principal components (PC1 and PC2) of the dataset, colored according to the original cell identities. (**F**) UMAP plot showing the first two components of the harmony-corrected dataset, colored according to the original cell identities.

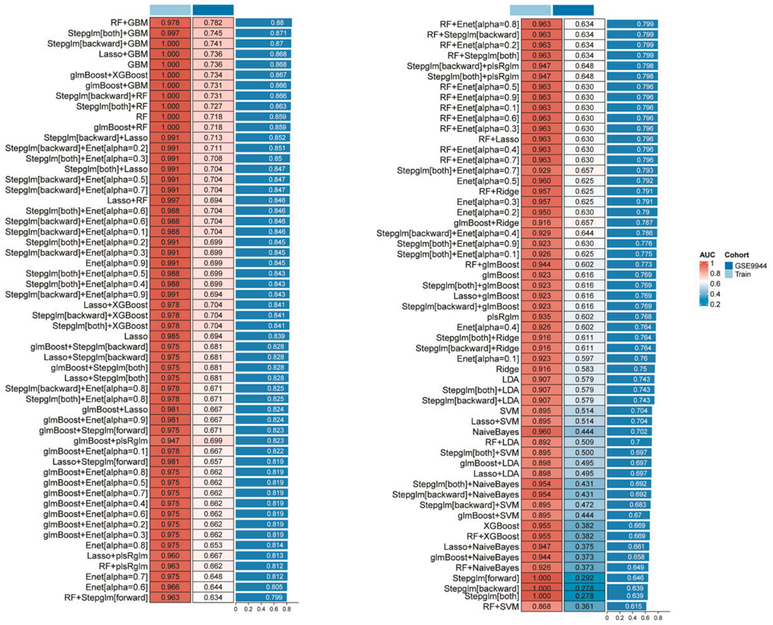

Supplementary Figure S2. Machine learning model performance evaluation. AUC values for different machine learning models, comparing the training cohort and external cohort (GSE9944). The performance of each model is represented by the AUC (area under the curve), with higher values indicating better model performance. The models are grouped based on their performance, with RF+GBM achieving the highest AUC (0.978) in the training cohort, and several other models showing good performance across both cohorts.
